# Symptoms of Depression, Anxiety, and Posttraumatic Stress among Patients with Cardiac Pacemakers

**DOI:** 10.3390/ijerph192416838

**Published:** 2022-12-15

**Authors:** Britta S. Bürker, Randolf I. Hardersen, Knut Tore Lappegård

**Affiliations:** 1 Department of Psychiatry, Nordland Hospital Trust Bodø, 8092 Bodø, Norway; 2 Norwegian National Unit for Sensory Loss and Mental Health, Oslo University Hospital, 0424 Oslo, Norway; 3 Department of Nephrology, Nordland Hospital Trust Bodø, 8092 Bodø, Norway; 4 Department of Clinical Medicine, UiT The Arctic University of Norway, 9037 Tromsø, Norway; 5 Division of Medicine, Department of Cardiology, Nordland Hospital Trust Bodø, 8092 Bodø, Norway

**Keywords:** anxiety, artificial pacemaker, coronary balloon angioplasty, depression, posttraumatic stress disorders, renal dialysis, patients and public health, social implications

## Abstract

Despite being a prerequisite for tailoring specific therapeutic interventions, knowledge of pattern and prevalence of clinically significant psychiatric symptomatology among patients with cardiac pacemakers (PMs), especially of symptoms of posttraumatic stress, is limited. We studied symptoms of depression, anxiety, and posttraumatic stress among PM patients (PM due to syncope or presyncope) compared to participants of (i) a cardiac, (ii) a chronic disease, and (iii) a healthy control group. Symptoms of depression, anxiety and posttraumatic stress were measured by validated self-report scales at least 6 months after implantation of the PM (PM group; *n* = 38), percutaneous coronary intervention (PCI; PCI control group; *n* = 23), and first dialysis (Dialysis control group; *n* = 17). Blood donors constituted the Healthy control group (*n* = 42). Both PM, PCI, and dialysis patients reported depressive symptoms above clinical cut-off more frequently than the healthy controls (16.2, 26.1, 41.2, and 0%, respectively; *p* < 0.001). Self-report of symptoms of anxiety and posttraumatic stress did not differ significantly across study groups. However, a non-negligible proportion of PM patients reported on symptoms of posttraumatic stress of anticipated clinical relevance. Identification and treatment of depression deserves attention in clinical routine in all three patient populations. Further study of posttraumatic stress in PM patients seems advisable.

## 1. Introduction

Cardiac pacemakers (PMs) are frequently implanted. In Norway in 2019, 699 new PMs per million inhabitants were implanted and approximately 28000 PM patients were followed up for their devices [1]. The majority of the PM recipients was aged > 60 years at the time of implantation, with 50% of recipients aged 61–80 years and approximately 40% of recipients aged 81 years or older [1]. A total of 88% of recipients received their PM due to syncope/dizziness/bradycardia or cardiac arrest [1].

Existing data indicate that adult patients with implantable cardiac devices, such as PM and implantable cardioverter-defibrillator, frequently report on symptoms of depression and anxiety [2,3]. For example, the mean baseline scores on the depression and anxiety subscales of the Hospital Anxiety and Depression Scale in a Chinese intervention study indicate that self-reported symptom levels of depression and anxiety among PM recipients are well above those reported in community-based samples [4,5].

Posttraumatic stress disorder (PTSD) is a psychiatric disorder with onset after a stressful life event and characterized by a set of specific symptoms, such as persistent remembering of the stressful life event (by intrusive flashbacks, vivid memories, or recurring dreams), avoidance of stimuli that can reactivate the memories, and hyperarousal [6]. PTSD is often accompanied by different anxiety symptoms [6] and is therefore often regarded as a form of anxiety disorder. However, PTSD necessitates a somewhat different therapeutic approach compared to other anxiety disorders and it is therefore important to diagnose PTSD specifically. While existing data indicate that a substantial portion of patients with implantable cardioverter-defibrillator report on symptoms of posttraumatic stress [7], to the best of our knowledge there is a lack of data on the occurrence of such symptomatology among PM patients. As a substantial proportion of PMs are implanted acutely in the aftermath of situations potentially perceived as life-threatening by the recipient (such as after a syncope or presyncope), and as the implantation of and the accommodation to a cardiac device further might be perceived as stressful life events, symptoms of posttraumatic stress have to be expected among PM patients as well.

Symptoms of depression, anxiety and posttraumatic stress/PTSD are not only associated with impaired quality of life, but these symptoms might also be associated with increased morbidity and mortality [8,9].

In order to tailor specific psychiatric/psychologic therapeutic interventions for PM patients, we need to increase our knowledge of the pattern and prevalence of clinically significant psychiatric symptomatology, especially of symptoms of posttraumatic stress/PTSD. Such specific interventions, when proven effective, will have the potential to attenuate cardiac intervention-related impairment of quality of life and might also impact positively on morbidity and mortality.

In this pilot study we aimed to explore the prevalence of symptoms of depression, anxiety, and posttraumatic stress measured by self-report among PM patients, who received their PM due to syncope or presyncope. We aimed further to compare the PM group’s symptom levels to those reported by participants of three control groups: (i) a cardiac control group, (ii) a chronic disease control group with known elevated occurrence of psychiatric symptomatology, and (iii) a healthy control group.

## 2. Materials and Methods

### 2.1. Study Sample

This pilot study was conducted at Nordland Hospital Trust Bodø (Norway) and included four study groups: PM patients, who had received a PM due to syncope or presyncope (PM group), patients who had received a percutaneous coronary intervention (PCI), patients receiving dialysis due to end-stage renal disease, and blood donors. The latter three study groups representing a cardiac control group (PCI control group), a chronic disease control group with known elevated occurrence of psychiatric symptomatology [10] (Dialysis control group), and a healthy control group (Healthy control group), respectively.

The study material was mailed to 52 eligible PM patients, 50 eligible PCI patients, and 50 eligible dialysis patients. Response rates were 73.1, 46.0, and 34.0%, respectively, resulting in the inclusion of 38 participants in the PM group, 23 participants in the PCI control group, and 17 participants in the Dialysis control group. Furthermore, 42 blood donors returned answered study material, constituting the Healthy control group. One additional individual returned the answered questionnaires, but this individual could not be included in the analyses due to missing data concerning study group.

#### 2.1.1. Inclusion Criteria

Participants in the PM group had received a PM due to syncope (defined as temporary loss of consciousness and muscle tone with complete recovery) or presyncope (defined as an episode of near-fainting). Participants in the PCI control group had received an elective or acute PCI due to stable or unstable angina pectoris or myocardial infarction. Participants in the Dialysis control group were treated with haemodialysis or peritoneal dialysis due to end-stage renal disease. Time of the index intervention (i.e., implantation of PM, performance of PCI, and first dialysis, respectively) antedated inclusion with at least 6 months. We chose this strategy in order to include participants with persistent psychiatric symptomatology as opposed to including in addition participants with transient difficulties with psychological adjustment to the intervention and its consequences. Participants in the Healthy control group were blood donors aged 50 years or older. We applied this age criterion as the PM, PCI, and dialysis patients were expected to be older than the majority of the blood donors at our blood bank.

#### 2.1.2. Exclusion Criteria

PM, PCI, and dialysis patients were not eligible for inclusion into the study if serious chronic somatic illness, cognitive impairment, and/or insufficient language skills (Norwegian) were documented in the electronic health record. In addition, we applied specific exclusion criteria regarding PM, PCI, and dialysis. Eligible PM and PCI patients had not received one of the two other interventions after the index intervention. By way of example, an eligible PM patient had not received a PCI after implantation of the PM nor had this patient started on dialysis after implantation of the PM. Eligible dialysis patients had not received a PM nor a PCI during the 6 months antedating inclusion.

For the participants of the Healthy control group no formal exclusion criteria were applied, as inclusion criteria for blood donors are strict.

### 2.2. Procedures

Eligible cardiac and dialysis patients were identified via electronic health records by a cardiologist (K.T.L.) and a nephrologist (R.I.H.), working at the Departments of Cardiology and Nephrology at Nordland Hospital Trust Bodø, respectively. The presence of inclusion and absence of exclusion criteria was evaluated at this stage, based on information from the electronic health record. Information about the study, the study questionnaires as well as a pre-paid return envelope were then sent to these eligible patients. Personnel at the blood bank approached eligible blood donors while they were at the blood bank, and handed out the study material in case of interest to participate in the study. A reminder was sent to all eligible PM, PCI, and dialysis patients to increase the response rate. Starting date for dispatch of study material was 16 March 2021 and the reminder was dispatched on 8 July 2021. The data base was closed on 27 September 2021.

Data was punched manually into the research data base, which was stored on a designated secure section of the server of Nordland Hospital Trust Bodø.

### 2.3. Measures

#### 2.3.1. Self-Report of Symptoms of Depression, Anxiety, and Posttraumatic Stress

Symptoms of depression, anxiety, and posttraumatic stress were measured by means of validated self-report scales, namely the Patient Health Questionnaire—9 (PHQ-9; [11]), the Generalized Anxiety Disorder—7 scale (GAD-7; [12]), and the Impact of Event Scale—Revised (IES-R; [13]).

The PHQ-9 is a 9-item self-report measure of depression severity [11]. Its items are based on the diagnostic criteria of the fourth edition of the Diagnostic and Statistical Manual of Mental Disorders (DSM-IV) [11]. Each item is rated from 0 to 3. The total score ranges from 0 to 27 with higher scores indicating more depressive symptoms. For a total score of 10 or higher, a sensitivity of 88% and a specificity of 88% to diagnose major depressive disorder has been reported for a sample of 580 patients, recruited into a primary care study [11]. A Cronbach’s α of 0.839 in the present sample indicates good internal consistency for the PHQ-9.

The GAD-7 is a 7-item self-report measure, developed to identify probable cases of generalized anxiety disorder and to assess symptom severity [12]. The final version of the GAD-7 is developed based on 9 items reflecting the DSM-IV diagnostic criteria for generalized anxiety disorder and 4 items based on review of existing anxiety scales [12]. Each item of the GAD-7 is rated from 0 to 3. The total score ranges from 0 to 21 with higher scores indicating more anxiety symptoms. The GAD-7 is correlated with two other self-report anxiety measures, namely the Beck Anxiety Inventory (r = 0.72) and the anxiety subscale of the Symptom Checklist—90 (r = 0.74) [12]. The GAD-7 is increasingly used as a measure of anxiety in general [14]. A total score of 8 has been proposed as threshold for identifying possible cases with anxiety disorder [14]. A Cronbach’s α of 0.897 in the present sample indicates good internal consistency for the GAD-7.

The IES-R is a 22-item self-report measure of symptoms of posttraumatic stress/PTSD [13]. Each item is rated from 0 to 4 with higher scores indicating more distress [13]. The items parallel the DSM-IV diagnostic criteria for PTSD [13]. Responses are reported as mean for 3 subscale scores (based on the respective single item ratings), namely the intrusion (8 items), the avoidance (8 items), and the hyperarousal (6 items) subscale [13]. For a valid self-report of symptoms of posttraumatic stress/PTSD by means of the IES-R, the respondent needs to relate the responses to a specific stressful life event [13]. Therefore, the stressful life event was explicitly stated on the questionnaire, namely the PM implantation (PM group), the PCI (PCI control group), and the first dialysis (Dialysis control group), respectively. The IES-R was not administrated to the Healthy control group. When evaluating the IES-R scores, the application of cut-offs is deemed inappropriate and this notion is discussed comprehensively elsewhere [13]. A Cronbach’s α of 0.880, 0.877 and 0.848 in the present sample for the intrusion, avoidance, and hyperarousal subscales, respectively, indicate good internal consistency for the subscales of the IES-R.

#### 2.3.2. Sociodemographic Variables

In this pilot study, only a limited number of sociodemographic variables was registered, namely gender, age categorized in 5-year intervals (i.e., <50, 50–54 years, etc.), study group (i.e., PM group, PCI control group, Dialysis control group, Healthy control group), and time since intervention categorized in 12-month intervals (i.e., 6 to <18 months, 18 to <30 months, etc.). For participants in the PM group, it was in addition registered whether they received their PM due to syncope or presyncope.

### 2.4. Statistical Analyses

For descriptive purposes, data are presented as frequencies and proportions or as means and standard deviations as appropriate. Symptoms of depression, anxiety, and posttraumatic stress were compared between study groups with the Kruskal–Wallis test (continuous variables) and when appropriate in addition with the Fisher exact test (i.e., PHQ-9 and GAD-7 total scores dichotomized at clinical cut-off; categorial variables). In case of significant group differences, we report results from posthoc analyses (pairwise comparisons) after Bonferroni correction. Level of significance was set to 5%. Statistical analyses were conducted with IBM SPSS Statistics software (IBM Corporation, Armonk, NY, USA) version 27.

## 3. Results

### 3.1. Sample Characteristics

**Figure 1:**
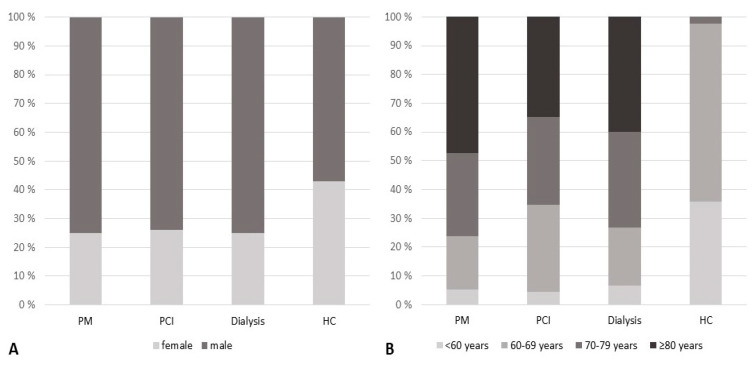
Gender and age distribution (*n* = 120 in total study sample). Panel (**A**) illustrates the gender distribution. Panel (**B**) illustrates the age distribution. Abbreviations: Dialysis: Dialysis control group. HC: Healthy control group. PCI: Percutaneous coronary intervention (PCI) control group. PM: Pacemaker (PM) group.

In the total study sample, 31.6% of participants were female. Age distribution in the total sample was as follows: 16.1% were younger than 60 years, 36.4% were between 60 and 69 years, 20.3% were between 70 and 79 years, and 27.1% were 80 years or older. Gender and age distribution in the Healthy control group were as expected somewhat different compared to the PM group, PCI control group, and Dialysis control group: Gender distribution tended to be more balanced in the Healthy control group compared to the three other study groups, and more participants in the Healthy control group were in the lower age categories compared to the three other study groups. Gender and age distribution are illustrated in Figure 1. Sociodemographic characteristics, including details on time since intervention, are depicted in Table 1.

### 3.2. Symptoms of Depression, Anxiety, and Posttraumatic Stress

**Figure 2:**
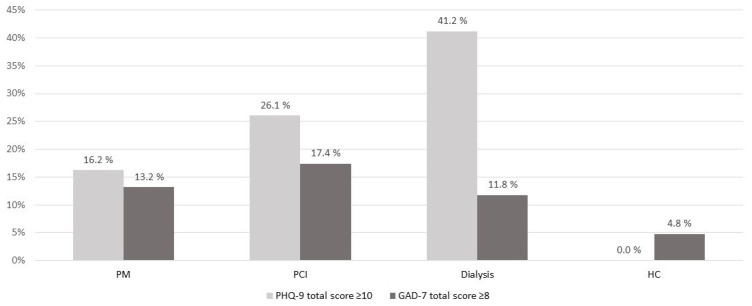
Proportion of participants with self-report of clinically significant depression and anxiety. Clinically significant depression is defined as PHQ-9 total score ≥ 10. Clinically significant anxiety is defined as GAD-7 total score ≥ 8. Clinically significant depression: *p* < 0.001 across study groups with the following statistically significant posthoc pairwise group comparisons after Bonferroni correction: PM vs. HC, PCI vs. HC, and Dialysis vs. HC. Clinically significant anxiety: *p* = 0.36 across study groups. Abbreviations: Dialysis: Dialysis control group. GAD-7: Generalized Anxiety Disorder—7 scale. HC: Healthy control group. PCI: Percutaneous coronary intervention (PCI) control group. PHQ-9: Patient Health Questionnaire—9. PM: Pacemaker (PM) group.

Mean PHQ-9 total scores differed significantly across study groups with the lowest group mean for the Healthy control group and the highest group mean for the Dialysis control group. Posthoc analyses revealed (after Bonferroni correction) statistically significant group differences between the PM group and the Dialysis control group as well as between the Dialysis control group and the Healthy control group, but no other statistically significant group differences in the remaining four pairwise comparisons. Further details are shown in Table 2. The proportion of participants with above cut-off self-report of depressive symptoms (i.e., PHQ-9 total score ≥ 10), and thus indicating the proportion of participants with clinically significant depression, differed also across study groups. This proportion was 16.2% in the PM group, 26.1% in the PCI control group, and 41.2% in the Dialysis control group, while none of the healthy control participants reported depressive symptoms above clinical cut-off. Posthoc analyses revealed (after Bonferroni correction) statistically significant group differences between the PM group and the Healthy control group, the PCI control group and the Healthy control group, as well as the Dialysis control group and the Healthy control group, but no other statistically significant group differences in the remaining three pairwise comparisons. Further details are shown in Figure 2.

The study groups did not differ significantly regarding self-report of symptoms of anxiety (measured by GAD-7) or posttraumatic stress (measured by IES-R). Neither differed the proportion of above cut-off self-report of anxiety symptoms across study groups. Details are depicted in Table 2 and Figure 2. Proportions of participants with IES-R subscale scores ≥ 2 (i.e., proportions of participants reporting that they on average at least were ‘moderately’ distressed regarding the items of the respective subscale) were in general low, ranging from 0 to 7.9% across study groups and subscales. The highest proportion (i.e., 7.9%) was found within the PM group for the intrusion and the hyperarousal subscales, followed by a proportion of 4.3% within the PCI control group for the avoidance and the hyperarousal subscales.

## 4. Discussion

We aimed to extend the knowledge about occurrence of symptoms of depression, anxiety, and posttraumatic stress measured by self-report among PM patients, and compare these findings to those in a cardiac control group, a chronic disease control group with known elevated occurrence of psychiatric symptomatology, and a healthy control group.

The study groups differed on the group level with regard to depressive symptoms, both when analysing data as continuous as well as dichotomous variable. Posthoc analyses indicated, that the proportion of participants with clinically significant depression (defined as above cut-off self-report) was higher among both the PM, PCI, and dialysis patients compared to the healthy control participants. Occurrence of clinically significant depression tended to be lower among PM patients (16.2%), than among PCI and dialysis patients, with the highest occurrence among dialysis patients (41.2%). A higher proportion of PM patients than healthy control participants with above cut-off self-report of depressive symptoms is in line with the results of a recently published cross-sectional study [15]. Due to differences in such as assessment methods and applied cut-off as well as differences in sample characteristics, it is difficult to compare previously published prevalence rates of clinically significant depression among PM patients directly to our findings. However, the proportion of PM patients with clinically significant depression included in our study (16.2%) compares fairly well with the absolute figures reported for above cut-off self-report of depressive symptoms in two earlier reports [16,17]. The proportion of PM patients with clinically significant depression in our study also seems higher than the sum of the 12-month prevalence for major depressive disorder and dysthymia (5.3%) reported from a Norwegian epidemiological, diagnosis-based study conducted in a rural area [18]. Relating these epidemiological data [18] to our findings in the Healthy control group indicates that our healthy control participants might be positively selected. Our results concerning self-report of depressive symptoms among the cardiac control group and the chronic disease control group seem in concordance with published data. The proportion of PCI patients with clinically significant depression (26.1%), compares well with the prevalence rate of depression mentioned in a review on depression among patients with cardiac disease (primarily based on data of patients with coronary artery disease) [19]. Our finding, indicating that 41.2% of the included dialysis patients were clinically depressed, is in line with what is reported in a review on depression among dialysis patients (i.e., 39.3% clinically depressed when depression was measured by self- or clinician-administered rating scales) [20].

The study groups did not differ statistically significant with regard to anxiety symptoms, neither when conducting the analysis with continuous nor with dichotomous variable. However, the proportion of PM, PCI, and dialysis patients with clinically significant anxiety (defined as above cut-off self-report) tended to be higher than the proportion of healthy control participants with clinically significant anxiety (11.8 to 17.4 vs. 4.8%). The 12-month prevalence for the specific anxiety disorders which were assessed in the aforementioned Norwegian epidemiological diagnosis-based study ranged from 1.1 to 5.0% (GAD vs. both specific phobia and social phobia, respectively) [18]. The 12-month prevalence for at least one anxiety disorder would be lower than the sum of the prevalence rates of the specific anxiety disorders (i.e., 13.9%) due to occurrence of comorbidity, but well above 5.0%. Contrasting these figures to our results indicates again that our healthy control participants might be positively selected. Even if a direct comparison is difficult, as outlined earlier, it seems as if above cut-off self-report of anxiety symptoms among included PM patients in our study (13.2%) is, in contrast to our expectations, somewhat lower than reported of others (i.e., 19.4 to 27.2%) [15,16,17]. Factors such as sociodemographic differences of the study samples (i.e., gender and age distribution), time since intervention, and recruitment strategies might contribute to these inconsistencies of results. The 17.4% of PCI participants with above cut-off self-report of anxiety symptoms in our study seems consistent with the notion in a review on anxiety disorders and cardiovascular disease, stating that 20 to 30% of patients experience elevated levels of anxiety following an acute coronary syndrome and that in half of cases anxiety persists for up to one year after the event [21]. Anxiety in dialysis patients seems less studied and reported prevalence rates vary greatly, ranging from 12 to 52% among haemodialysis patients [22]. Prevalence rate of anxiety disorders in a Norwegian diagnosis-based study in dialysis patients was 17% [23], compared to the 11.8% of dialysis patients with above cut-off self-report of anxiety symptoms in our study.

Symptom levels of posttraumatic stress did not differ statistically significant between PM, PCI, and dialysis patients in our study. However, it is noteworthy that the proportions of participants reporting on average at least ‘moderate’ distress on two of the three subscales of the IES-R were highest in the PM group. To the best of our knowledge, posttraumatic stress/PTSD has not been studied specifically in PM patients earlier. A 12% prevalence rate of PTSD secondary to acute coronary syndromes is mentioned in a review [24], thus indicating that the level of self-report of posttraumatic stress among the included PCI patients in our study is lower. This inconsistency might partly be explained by the fact that our PCI control group included patients with both acute and elective PCI. Prevalence rate of PTSD related to haemodialysis was 10.4% in a cross-sectional study [25]. This indicates that PTSD occurred more frequently in that study than among dialysis patients included in our study. This inconsistency can hardly be explained through differences in sociodemographic characteristics and assessment methods alone, and remains thus unexplained.

### Limitations

Our pilot study has several limitations that need to be discussed. First, we included only participants in the PM group that had received their PM due to syncope or presyncope. Thus, our results are not necessarily generalizable to all PM patients. However, at least the age distribution of the participants in the PM group seems to resemble the Norwegian population of PM recipients [1]. Second, the exclusion criteria differed slightly between the PM group, PCI control group, and the Dialysis control group. While PM and PCI patients were excluded if they had received one of the two other interventions after the index intervention (in order to be able to demonstrate the potential effect of the intervention in question), dialysis patients were only excluded if they had received a PM or a PCI during the 6 months antedating inclusion. This adaption was necessary to avoid selection bias in the Dialysis control group due to the higher occurrence of comorbidity in the dialysis population. This minor difference in exclusion criteria might have influenced on our results. Third, even though we excluded patients for whom cognitive impairment was documented in the electronic health record, we must expect that some of our included patients might have some degree of cognitive impairment (due to for example advanced age), which might have influenced on self-report and thus our results. Fourth, our participants were not diagnosed after a structured psychiatric interview of an experienced clinician (which is the gold standard), which makes it for example difficult to draw firm conclusions regarding the lower-than-expected occurrence of above cut-off self-report of anxiety symptoms among the PM patients in our study. Fifth, the response rates varied between the eligible PM, PCI, and dialysis patients, with a response rate as high as 73.1% in the PM group and as low as 34.0% in the Dialysis control group. Against the background that psychiatric symptomatology seems to occur most frequently among dialysis patients and that patients with psychiatric symptomatology might be less prone to participate in a study like ours, the occurrence of symptoms of depression, anxiety, and posttraumatic stress might be underestimated in our study, especially among the included dialysis patients. Sixth, as prevalence rates of depression and anxiety disorders in general are higher in females than in males [18], it would have been interesting to re-analyse our data controlling for gender. However, we deemed our sample too little to conduct these analyses. In the same line, it would have been interesting to analyse the effect of age (especially with regard to self-report of depressive symptoms) and time since intervention on the occurrence of symptoms of depression, anxiety and posttraumatic stress.

## 5. Conclusions

At 6 months or more after the intervention, the included PM, PCI, and dialysis patients reported more frequently on depressive symptoms above clinical cut-off than the healthy control participants. Furthermore, the occurrence of above cut-off self-report of depressive symptoms in these patient groups seems to be higher than expected in the general population. Thus, even if clinically significant depression seems to occur more frequently among dialysis patients than among PM and PCI patients, it seems necessary to focus on identifying relevant patients in all three patient populations in clinical routine. Patients with depressive symptomatology of suspected clinical relevance should be referred to a mental health professional for adequate diagnostic evaluation and treatment, with the aim to increase quality of life and possibly contribute to alleviate depression’s negative impact on clinical outcomes. It is, as outlined above, difficult to draw firm conclusions of our findings regarding anxiety symptoms in the PM group. However, together with our finding that a non-negligible proportion of PM patients reported on symptoms of posttraumatic stress of anticipated clinical relevance, it seems advisable with further studies in the field. These studies should in a first step aim to establish stable estimates of prevalence of PTSD among PM patients, based on the diagnostic criteria of the latest (i.e., fifth) version of the Diagnostic and Statistical Manual of Mental Disorders, as well as of posttraumatic stress symptoms of insufficient severity to render a formal diagnosis (i.e., sub-diagnostic symptom level).

## Figures and Tables

**Table 1:** Sociodemographic characteristics of the study sample.

	PM		PCI		Dialysis		HC		Total Sample	
***n***	38 ^a^		23		17		42		120	
**Sex, f/m (% f) ^b^**	9/27	(25.0)	6/17	(26.1)	4/12	(25.0)	18/24	(42.9)	37/80	(31.6)
**Age, *n* (%) ^c^**										
<70 years	9	(23.7)	8	(34.8)	4	(26.7)	41	(97.6)	62	(52.5)
≥70 years	29	(76.3)	15	(65.2)	11	(73.3)	1	(2.4)	56	(47.5)
**Time since intervention ^d^,** ***n* (%) ^e^**										
6 to <18 months	24	(63.2)	22	(95.7)	9	(60.0)				
18 to <30 months	14	(36.8)	1	(4.3)	1	(6.7)				
30 to <42 months	-	-	-	-	5	(33.3)				

Abbreviations: Dialysis: Dialysis control group. f: Female. HC: Healthy control group. m: Male. PCI: Percutaneous coronary intervention (PCI) control group. PM: Pacemaker (PM) group. Annotations: ^a^ Indication for PM: *n* = 27: syncope; *n* = 11: presyncope. ^b^ Missing value for *n* = 2 of PM and *n* = 1 of Dialysis. ^c^ Missing value for *n* = 2 of Dialysis. ^d^ Interventions defined as: PM: implantation of PM due to syncope or presyncope; PCI: elective or acute PCI due to stable or unstable angina pectoris or myocardial infarction; Dialysis: first dialysis due to end-stage renal disease. ^e^ Missing value for *n* = 2 of Dialysis.

**Table 2:** Self-report of symptoms of depression, anxiety, and posttraumatic stress (measured by PHQ-9, GAD-7 and IES-R, respectively) according to study group.

	PM		PCI		Dialysis		HC		*p*
**PHQ-9 total score, M (SD) ^a^**	4.1	(5.2)	5.4	(5.0)	7.3	(4.9)	2.6	(2.4)	0.002 ^b^
**GAD-7 total score, M (SD)**	2.6	(4.6)	3.3	(3.7)	3.1	(3.0)	1.4	(2.4)	0.08
**IES-R ^c^**									
Intrusion subscale, M (SD) ^d^	0.4	(0.7)	0.3	(0.4)	0.5	(0.5)			0.33
Avoidance subscale, M (SD) ^e^	0.3	(0.5)	0.3	(0.7)	0.4	(0.4)			0.51
Hyperarousal subscale, M (SD)	0.4	(0.7)	0.4	(0.6)	0.4	(0.4)			0.37

Abbreviations: Dialysis: Dialysis control group. GAD-7: Generalized Anxiety Disorder—7 scale. HC: Healthy control group. IES-R: Impact of Event Scale—Revised. M: Mean. PCI: Percutaneous coronary intervention (PCI) control group. PHQ-9: Patient Health Questionnaire—9. PM: Pacemaker (PM) group. SD: Standard deviation. Annotations: ^a^ Missing value for *n* = 1 of PM. ^b^ The following posthoc pairwise group comparisons were statistically significant after Bonferroni correction: PM vs. Dialysis and Dialysis vs. HC. ^c^ The IES-R was not administered to HC. ^d^ Missing value for *n* = 1 of Dialysis. ^e^ Missing value for *n* = 2 of PM.

## Data Availability

The data presented in this study are available on request from the corresponding author within the limits of applicable Norwegian law.
